# Assessment of Pristine Carbon Nanotubes Toxicity in Rodent Models

**DOI:** 10.3390/ijms232315343

**Published:** 2022-12-05

**Authors:** Marta Witkowska, Ewa Florek, Radosław Mrówczyński

**Affiliations:** 1 Faculty of Chemistry, Adam Mickiewicz University, 61-614 Poznań, Poland; 2 Centre for Advanced Technologies, Adam Mickiewicz University, 61-614 Poznań, Poland; 3 Laboratory of Environmental Research, Department of Toxicology, Poznan University of Medical Sciences, 30 Dojazd, 60-631 Poznań, Poland

**Keywords:** nanotoxicity, toxic, carbon nanotube, animal model, rodent, pristine

## Abstract

Carbon nanotubes are increasingly used in nanomedicine and material chemistry research, mostly because of their small size over a large surface area. Due to their properties, they are very attractive candidates for use in medicine and as drug carriers, contrast agents, biological platforms, and so forth. Carbon nanotubes (CNTs) may affect many organs, directly or indirectly, so there is a need for toxic effects evaluation. The main mechanisms of toxicity include oxidative stress, inflammation, the ability to damage DNA and cell membrane, as well as necrosis and apoptosis. The research concerning CNTs focuses on different animal models, functionalization, ways of administration, concentrations, times of exposure, and a variety of properties, which have a significant effect on toxicity. The impact of pristine CNTs on toxicity in rodent models is being increasingly studied. However, it is immensely difficult to compare obtained results since there are no standardized tests. This review summarizes the toxicity issues of pristine CNTs in rodent models, as they are often the preferred model for human disease studies, in different organ systems, while considering the various factors that affect them. Regardless, the results showed that the majority of toxicological studies using rodent models revealed some toxic effects. Even with different properties, carbon nanotubes were able to generate inflammation, fibrosis, or biochemical changes in different organs. The problem is that there are only a small amount of long-term toxicity studies, which makes it impossible to obtain a good understanding of later effects. This article will give a greater overview of the situation on toxicity in many organs. It will allow researchers to look at the toxicity of carbon nanotubes in a broader context and help to identify studies that are missing to properly assess toxicity.

## 1. Introduction

Carbon nanotubes (CNTs) have a high surface-to-weight ratio, which increases surface reactivity on nanotubes that can enter through the pores or through the lipid bilayers [1]. They can take various shapes, ranging from fibrous to spherical, which influences their properties and biological activity [2]. Due to their unique mechanical, optical, electrical, and chemical properties, CNTs have been used in medicine and pharmacy studies, including sensitive detection of key biological molecules, more accurate and safe imaging of diseased tissues, and innovative forms of therapy [3].

Carbon is one of the most versatile elements on the periodic table due to the variety of possible bonds between carbon and other elements. The bond’s strength between carbon and many elements is also important. The ability of carbon orbitals to hybridize in sp, sp2, and sp3 configurations allows for the existence of allotropic varieties, which include naturally occurring diamond, amorphous carbon, and graphite, as well as synthetic graphene, carbon nanotubes, fullerenes, or nanodiamonds [4]. Carbon nanotubes were discovered by Sumio Iijima in 1991, who noticed fibers with a diameter of several nanometers and a length of several micrometers made of carbon atoms [5]. CNTs are modified nanoparticles formed by graphene, which can be a single folded sheet, called single-walled (SWCNT), or a concentric multiple sheet, called multi-walled (MWCNT). Ideal CNTs have all carbon atoms bound in a hexagonal grid except for their ends. There are defects in mass-produced carbon nanotubes, causing imperfections in the sidewalls that impair the desired properties of these nanoparticles. SWCNT and MWCNT diameters range from 0.8 to 2 nm and 5 to 20 nm, although polyhedral diameters can exceed 100 nm, which mainly depends on the number of layers of nanotube walls and functional groups attached to it. Most SWCNTs have a diameter of close to 1 nm and they exhibit important electric properties, which do not occur in MWCNTs [6]. Size is an important property of carbon nanotubes, because the reduction in size results in an increase in the particle surface area. This allows for more molecules to bind to the surface area, causing an increase in toxic effect [7]. A characteristic feature of carbon nanotubes is a much greater reactivity than graphene, which results from the existence of defects in the lattice formed by carbon atoms. The ends of nanotubes are generally more chemically reactive than their flat surface. A useful and currently used phenomenon is the ability of nanotubes to attach various atoms using covalent bonds on their surface. Thanks to this, it is possible to obtain materials with the desired properties [8]. What makes carbon-based nanomaterials of great interest is also their unique chemical and physical properties, including thermal, mechanical, electrical, and optical [9].

Additionally, CNTs exhibit an elongated structure, high mechanical strength, high electrical conductivity, high thermal conductivity, metallic or semi-metallic behavior, and a large surface area, as well as being ultra-light weight [10]. Due to their nanometric size and high drug loading efficiency, CNTs are preferable in cancer therapy studies. CNTs are used to deliver drugs or other small therapeutic molecules to target sites, and they are also valuable in photothermal and photodynamic therapy, as well as in combination therapies [11]. The small size of CNTs allows them to enter all types of cells, including mammalian, yeast, and bacterial cells [12]. Their needle-like shape and high surface area allow for adsorption or conjugation with various therapeutic molecules [13]. The common uses of CNTs for the delivery of anticancer drugs include conjugating topoisomerase inhibitors, platinum (Pt)-based drugs, and antimicrotubules to the material of interest, allowing them to penetrate together [13,14]. In addition, biodistribution studies have shown that functionalized SWCNTs accumulated in the tumor, which has resulted in them being frequently investigated for the delivery of anticancer agents [15]. Antitumor effect has been proven in many in vivo studies [16,17,18,19,20]. CNTs make an interesting vector for gene delivery, as they manage to enter the cell directly through the plasma membrane, due to their lipophilicity and size, without the use of endocytic pathways [21]. They have also been used in research for vaccine delivery for immunizing cancer and other infectious diseases. The basic concept of using carbon nanotubes in vaccine delivery is to combine an antigen with carbon nanotubes to maintain the correct conformation and, by that, induce a specific antibody response. In addition, CNTs are used as photothermal agents in photothermal therapy [22,23]. They strongly absorb light and convert its energy into hyperthermia, which results in the thermal ablation of adjoining cells [24]. However, the usage of CNTs as a carrier for drug and gene delivery or in advanced nanotherapies should not trigger an immune system response [25]. CNTs are unique due to their mechanical, electrical, and optical properties, as well as the possibility of filling them with various compounds, including drugs, which makes them a promising nanomaterial for biomedical applications. Carbon nanotubes can be used as efficient biocompatible biosensors, contrast agents for imaging, and can also extend the lifetime of drugs in the body and facilitate their direct delivery to cells. They are also useful in cell growth and recolonization, particularly in the case of nerve cells [26]. Research is emerging focusing on pristine and functionalized carbon nanotubes for biomedical applications, so it is important to understand their toxic effects thoroughly, and for this we need a unified procedure for the toxicological assessment of carbon nanotubes.

Thus, the information, especially from studies with rodents, regarding toxicity of CNTs, their accumulation in the body, and their side effects are of great importance for the development of the nanomedicine field. Most in vivo studies of carbon nanotubes use rodents as animal models [27,28]. Here, we summarize the latest reports dealing with toxicological studies of different CNTs exploiting only advanced animal models. The text is divided into six subchapters that present the impact of CNTs on the main organs, such as livers, kidneys, hearts, lungs, and brains. We also discuss the general toxicity of CNTs. Finally, in the conclusion section we outline the latest achievements in this field and give some perspective for further research. In reviewing the toxic effects of carbon nanotubes, we considered many aspects. CNT toxicity should be defined in terms of acute, subacute, subchronic, or chronic exposure conditions. Our evaluation covers in vivo toxic effects on various organs while considering different routes of exposure, doses, exposure time to CNTs, and their potential toxic mechanisms after administration of pristine carbon nanotubes in in vivo experiments in mice and rats. We believe these findings could provide an important benchmark for assessing and managing human risk after exposure to carbon nanotubes.

## 2. Toxicological Aspects

Carbon nanotubes have many advantages in the medical field. However, the properties that make them attractive to use may also affect their toxicological profile in biological systems, so their size, shape, and chemical composition must be taken into account in the production. Concerns also relate to the interaction of cellular networks, the endocytic pathway, and the absorption process, which can also induce cytotoxicity, leading to disturbances in cell homeostasis [29]. One of the key factors for interaction with the biological systems of CNTs is their size, which is strongly related to toxicological effects. Smaller particles have a larger surface area per unit mass and are therefore able to absorb a large number of chemical molecules. This results in increased reactivity in the cellular environment and thus, greater toxicological effects [7]. In vitro studies have shown that CNTs below 10 nm are potentially harmful to the lungs due to their large surface area and possible nuclear penetration [30].

The importance of nanotoxicity is that even when they are made of inert materials, they are very active due to their nanosize. Thus, nanotoxicity seeks to establish the level or extent to which these properties may pose a risk to the environment or life of organisms. The rationale for starting to design nanodrugs is to reduce the toxicity of the drug and increase its bioavailability and biocompatibility. On the other hand, it must be taken into account that their specific properties may pose a threat to patients. Carbon nanotubes exhibit toxicity through various mechanisms and can affect allergy, fibrosis and organ failure, neurotoxicity, hepatotoxicity, nephrotoxicity, and pulmonary toxicity [31].

Dose characterization of the carbon nanotubes is also an important aspect, and is also crucial for the interpretation of the results. Defining the dosage of CNTs, however, is complicated by the lack of data on specific doses and the effect of aggregation or stability on effective dosing and toxic dose. Some studies, however, indicate dose-dependent toxicity. Hojo et al. indicated in their research that high dosages of MWCNTs significantly increased incidences of lung carcinomas, lung adenomas, and pleural mesotheliomas [32].

The intrinsic toxicity of CNTs depends on the degree of surface functionalization and the functional groups presented in the CNTs. Another important factor of CNT toxicity is its bioavailability. Metabolism, degradation, dissolution, clearance, and bioaccumulation require attention and research to understand the limitations of CNTs as pharmaceuticals [33]. Carbon nanotubes act as haptens and change the structure of the protein, making it more antigenic, increasing the autoimmune effect. In vitro and in vivo studies of CNT toxicity are mainly conducted in mice and rats. The toxicity assessment includes a cell proliferation/viability test, an apoptosis detection test, a reactive oxygen species production test, and a measurement of superoxide dismutase. The mechanisms responsible for their toxicity are mainly oxidative stress, membrane damage, and genotoxicity [34]. Carbon nanotubes can also cause genotoxicity. Primary genotoxicity arises in the absence of an inflammatory response, while secondary genotoxicity is driven by the activation of inflammatory cells such as neutrophils and macrophages, which can produce significant amounts of reactive species in an oxidative burst [35]. CNTs can induce formation of ROS by interacting with cellular components such as mitochondria and cell membrane and are capable of attenuating intracellular antioxidant defense (AOX) [36,37]. Toxicity can also be caused by the presence of toxic catalyst residues that are necessary for CNT synthesis [38]. Studies have also shown that CNTs are cytotoxic with negative effects on the cardiovascular and reproductive systems. Despite all the features that make carbon nanotubes toxic, the functionalization strategies used reduce the toxic effects and increase their usefulness in biomedicine [39].

An important aspect of the toxicity of carbon nanotubes in biological systems includes absorption, distribution, metabolism, and elimination (ADME). As pristine CNTs are hydrophobic nanomaterials, they have great potential to form aggregates in blood systems. This problem can be solved by functionalizing carbon nanotubes, i.e., modifying their surface with different molecules obtained by adsorption, electrostatic interaction, or covalent bonding of different molecules, which makes them more hydrophilic, thus changing the profile of biocompatibility [33]. The fate of carbon nanotubes in biological systems is an important concern for future use in nanomedicine and pharmacy. The biodistribution and pharmacokinetics of CNTs depend strongly on their physicochemical characteristics, such as surface functionalization, solubility, shape, and aggregation. Carbon nanotubes that enter the body through inhalation have a very long pulmonary half-life, from months to even years. This means that extrapulmonary effects may appear long after exposure has ended [40]. Physiology suggests that when the particles are in the blood, there are two common excretion pathways, known as renal and hepatobiliary clearance. Small particles are often excreted in the urine, and larger ones tend to accumulate in the body, mainly in the Kupffer cells of the liver [41]. One hypothesis suggests that when CNTs are injected into the circulation through the caudal vein, the first organ to which they will go is the lungs [40]. As carbon nanotubes are meant to be used in humans, their metabolism is a critical issue. The metabolism of carbon nanomaterials in the human body is a critical issue if carbon nanotubes are to be used as a human biological imaging agent or drug carrier. If CNTs are not degraded or entirely eliminated from the body, they may accumulate in organs [42]. Recent studies have shown that carbon nanotubes can be degraded by enzymes such as horseradish peroxidase [43] and myeloperoxidase (MPO) [44], macrophages [45], and neutrophils [46].

Mice and rats are widely used in nanotoxicity studies. A large suite of biomarkers, such as clinical pathology and chemistry, organ and body weights, and immunogenicity and microscopic evaluation of tissues, can be used to provide quantitative information about the biological state of animal models, and as a consequence, predictive human effects. Rats and mice are appropriate species for toxicological studies based on a comparison of the pharmacokinetics, target pharmacodynamics, and metabolism. In addition to their metabolic similarities, they are great choices for toxicological research thanks to their small size, short life span, and relatively easygoing nature [47]. Assessment of toxicity using rodent models includes tissue structural changes, apoptosis, and inflammation in major target organs and other systems susceptible to accumulation of carbon nanotubes [48]. There are a variety of methods used to evaluate the toxicity of carbon nanotubes, however, there are no standard methods, which makes it difficult to compare the results. The reason behind this may be a broad range of physicochemical properties that can influence the toxicity of CNTs.

## 3. Pulmotoxicity

Lungs are a pair of spongy, air-filled organs responsible for carrying out gas exchange, which includes oxygen uptake and carbon dioxide removal [49]. The respiratory tract is in continuous contact with the environment, by inhaling particles and waste matter. Therefore, the respiratory system is the most critical target for inhaled carbon nanotubes. Afterward, they are translocated to blood and distributed throughout the body [50]. Inhalation of CNTs is one of the most common methods of CNT delivery [51]. Particle size, functionalization, and dispersion contribute greatly to the development of pulmonary toxicity [52]. Various results indicated that the main cause of SWCNT toxicity may be the deposition of fine carbon nanotubes in the alveolar tissue and endotracheal wall. As they are not macromolecules, it has been found that they do not settle on the inner walls by themselves, but rather as a group of particles that can lead to tumor formation in the inner walls of the respiratory tract [53]. Due to their small size, carbon nanotubes can enter the lower airway, reach the alveoli, and pass the alveolar epithelium to the intrapleural space [54]. Particles may be exposed to various forces, such as inertia, gravity, or diffusion, and in the case of CNTs, diffusion tends to dominate. The size of the carbon nanotubes impacts their deposition, therefore, particles smaller than 5 nm are deposited in the nasopharyngeal region, while larger ones in the alveolar region [55]. These differences in CNT deposition may influence their biological effects. CNTs may reach interstitial sites, be taken up into the blood cells of the alveoli during oxygen exchange, and be readily translocated to extrapulmonary sites where they reach other target organs by different transfer routes and mechanisms. The extent of extrapulmonary translocation is highly dependent on particle surface characteristics and chemistry [56]. To test toxicity in lungs, carbon nanotubes were administrated by intratracheal instillation [57,58,59,60], pharyngeal aspiration [61,62], nose-only inhalation [63,64,65], whole-body inhalation [66], intratracheal intrapulmonary spraying [67], or intragastric administration [68]. Although carbon nanotubes can be administrated to the lungs in a different way, there are differences in the distribution, clearance, and retention of the materials in the lungs. Particle deposition and dimensions are major factors in the toxicological potential of inhalable materials, although, rodents are obligate nasal breathers and so only restricted amounts of particles can penetrate into the lungs throughout the inhalation [69]. The amount of particles that reach the lungs is uncertain, moreover, even with the same concentration of aerosolized fibers, the quantity of particles inhaled by humans will differ from the amount inhaled by rodents. Therefore, for the number of materials administrated into the lungs to be known, the better way is the use of intratracheal instillation, which allows direct injection of the particles into the trachea [58].

Hojo et al. studied the carcinogenic potential of multiwalled carbon nanotubes by intratracheal installation in their 2-year studies. In their studies, they used thick, long, and straight fiber MWNT-7, which was non-functionalized. They found that rats that had accumulated about 3.6 mg of MWCNT fibers in their lungs had an increased occurrence of lung and pleural tumors. Hojo et al. assessed toxicity by analyzing survival period, body and organ weights, histopathological analysis of non-neoplastic and neoplastic lesions, cytological and biochemical analyses of bronchoalveolar lavage fluid, as well as quantification and structural characterization of MWCNTs in the lung and pleural lavage fluid [32]. This research can be compared to the study by Kasai et al., which tested lung carcinogenicity of inhaled non-functionalized MWNT-7 in rats by using the same method for evaluation as Hojo et al. There was a confirmation that MWCNTs are carcinogenic to the lungs, however, no plural mesothelioma was observed compared to intratracheal instillation. [70] Evidence of mesotheliomas were shown in Hojo et al. and Kasai et al., both of which were long-term research. This may be due to the time needed for this symptom to appear, supporting the necessity for long-term research to understand the exact toxicity of carbon nanotubes, which can also be confirmed by Donaldson et al. [71]. Fujita et al. have analyzed pulmonary toxicity of carbon fibers of different diameter and length, and compared them to non-functionalized MWCNTs. CNTs were administered by intratracheal instillation, and then their general condition, weight gain, organ weight, bronchoalveolar lavage fluid (BALF) analysis, histopathological examination, and lung load were examined. As a result of CNT deposition in the lungs, black spots were observed. Fujita et al. recognized that the emergence of a sustained inflammation and pneumonia response was due to the deposition of carbon nanotubes, however, histopathological examinations and analysis of residual carbon nanotubes in the lungs and lymph nodes showed their gradual removal from the lungs [57]. In the studies of Numano et al., intratracheal administration of non-functionalized MWCNT-7 resulted in an increase in lung weight and an inflammatory response resulting in histopathological changes. The administration of carbon nanotubes also led to a delay in the parietal pleura. Toxicity was evaluated by measuring body and organ weight, food consumption, macroscopic pathological examination, lung burden, cytological analysis, and clinical chemistry in the BALF, along with histopathological examination [58]. Seidel et al. showed a toxicogenomic approach, which indicated that, despite their particular morphology, pristine MWCNTs induced lung inflammation and adjusted expression of genes and proteins. They also showed that even if exposure to carbon nanotubes induced a moderate influx of neutrophils, it balanced the expression of genes that could be involved in lung pathologies. This has been estimated by analyses of the transcriptome in the whole lung and the proteome in the bronchoalveolar lavage fluid of exposed animals [63]. Folkman et al. exposed SWCNTs to animals via gastric administration. This exposure route is particularly relevant to human health because CNTs can have directed or undirected potential for ingestion, and inhaled carbon nanotubes can be eventually removed via the gastrointestinal tract [72]. Moreover, potential exposures include consumption of fish or shellfish that have gathered CNTs due to ingestion or absorption of contaminated discharge [73]. The potential for different effects in the intestinal and extraintestinal compartment is shown in this study, using 0.064 and 0.64 mg/kg of pristine SWCNTs administered as a one-time dose to rats. Toxicity was assessed by measuring the level of oxidative damage to DNA as the premutagenic 8-oxo-7,8-dihydro-2 ’-deoxyguanosine (8-oxodG) in the colon mucosa, liver, and lungs. The results presented oxidative DNA damage in the liver and lungs, but not in the colon, which is likely due to genotoxic capacity rather than an inhibition of the DNA repair system [68]. Publications on the pulmotoxicity of pristine carbon nanotubes in rats and mice models are shown in chronological order in Table 1.

## 4. Hepatotoxicity

The liver is a multifaceted gland with various functions. Due to Kupffer cells, the liver is the main organ in charge of inborn immunity [76]. Kupffer cells are the most abundant macrophage population in the body and are directly in contact with blood by lining the wall of liver sinusoids. As part of the reticuloendothelial system, these macrophages are responsible for the capture of circulating carbon nanotubes and, therefore, constitute a highly suitable model to study carbon nanotube toxicity [77]. The hepatic sinuses, Kupffer cells, and the glomerular basement membrane are used for the metabolism and cleansing of the liver [78]. The liver accumulates 30–99% of administered CNTs from the bloodstream, leading to increased toxicity of hepatic cells [79]. The mechanisms of hepatotoxicity are oxidative stress and inflammation. ROS are formed as a by-product of normal oxygen metabolism and play an important role in cell signaling and homeostasis. CNTs directly induce the formation of ROS, causing oxidative damage to various cells, inducing inflammation, and inducing apoptosis. After administration of CNTs, they increase the level of free radicals in the cells [80]. ROS react with macromolecules in the cell, including proteins and lipids, disrupting homeostasis and activating specific oxidative stress signaling pathways such as activating protein-1 (AP-1), nuclear factor kB (NF-kB), and mitogen-activated protein kinase (MAPK). This leads to an increase in proinflammatory cytokines and chemokines, and also depletes antioxidants, causing exacerbation of inflammation [81]. These major mechanisms ultimately lead to DNA damage, cell death, gene mutation, and the malignant transformation of normal cells.

Carbon nanotubes can target the liver through pulmonary and systemic exposures [82]. Kim et al. and Lin et al. showed that administration of CNTs to animals by intratracheal instillation causes inflammation [74] and injury in the liver [83]. Moreover, in the studies where carbon nanotubes were administrated intravenously, the hepatotoxicity was revealed, which was caused by inflammation, oxidative damage, genotoxicity, or mitochondrial destruction [84,85,86,87]. Kim et al. used tracheal instillation to expose mice to pristine MWCNTs. One year effect of carbon nanotube administration was elucidated by histopathological analyses along with measurement of serum cholesterol homeostasis, and inflammatory, protein, and serum cytokine levels [74]. Lin et al. exposed rats to pristine SWCNTs via intratracheal instillation. Acute toxicity was then explored using metabonomic analysis of ^1^H NMR spectra of blood plasma and liver tissue extracts, and liver histopathology and measurements of biochemical indicators on rats’ liver functions. Results showed evident changes in clinical chemistry, indicating SWCNTs could cause hepatotoxicity through pathophysiologic necrosis and inflammation. ^1^H NMR spectroscopic and pattern recognition analysis of aqueous soluble liver extract revealed that SWCNTs could lead to cell oxidative damage [83]. Folkman et al., as mentioned earlier, looked into oxidatively damaged DNA of pristine C_60_ fullerenes and single-walled carbon nanotubes after oral administration. In the liver, there were increased levels of 8-oxodG, most likely caused by direct genotoxic ability [68]. In their study, Yang et al. intravenously administrated pristine SWCNTs, and after a 3-month period of time, they investigated hepatotoxicity in mice. To test toxicity of carbon nanotubes, they looked into clinical symptoms and organ indices, serum biochemical parameters, histopathological observation, serum immunological observations, cell apoptosis, and oxidative stress. Results showed low toxicity in mice only through serum biochemical changes. Yang et al. proposed oxidative stress in the liver as a main toxicological mechanism for hepatotoxicity [87] (Table 2).

## 5. Nephrotoxicity

Kidneys perform various functions in our body. They excrete the end products of metabolism, regulate the synthesis and release of hormones, and nephrons maintain fluid homeostasis, osmoregulation, and waste filtration [88]. Because it is the primary organ of excretion, we can suspect that carbon nanotubes will adversely affect the renal system [50]. Similarly to in hepatotoxicity, the nephrotoxicity, renal clearance, and excretion of carbon nanotubes depend on their aspect ratio, surface hydrophobicity, metallic impurity, and functionalization [89]. Kidneys of rats exposed to unfunctionalized and functionalized carbon nanotubes showed closely similar disruptions, which were probably induced by excess of reactive oxygen species and inflammation. Hence, certain negative properties, typical for carbon nanotubes such as cytotoxicity, poor blood compatibility, inflammatory effects, and target-organ toxicity are found also in functionalized particles [90]. Toxic effects of carbon nanotubes on animals’ kidneys are summarized in Table 3. Zamani et al. showed that pristine MWCNTs have the ability to enter the body, and eventually to cross cellular barriers and reach the kidney as a sensitive organ, which can result in mitochondrial damage in kidney cells. To test nephrotoxicity, they used a succinate dehydrogenase activity assay, mitochondrial ROS generation assay, mitochondrial membrane potential collapse evaluation, evaluation of mitochondrial swelling, and determination of cytochrome c release [91]. Awogbindin et al. researched hepatic and renal dysfunction after intravenously injecting multi-walled carbon nanotubes, one of which was a pristine MWCNT. To examine nephrotoxicity of carbon nanotubes, biochemical determination of indices of oxidative stress and damage was made, as well as using histological analyses. Tissue-specific levels of proinflammatory markers were also assessed. Urea and creatinine levels were reduced, and myeloperoxidase activity, nitric oxide level, reactive oxygen and nitrogen species, and tumor necrosis factor level in kidneys were increased [92]. Guzmán-Mendoza et al. took pristine, pre, and functionalized carbon nanotubes under investigation. MWCNTs were administrated intravenously, and biochemical and histopathological parameters were analyzed at 1, 14, 29, and 60 days post-exposure. Pristine MWCNTs have shown the highest toxicity, especially due to accumulation in the kidneys and the lungs even at 60 days [93]. Tang et al. studied short-term and long-term toxicity of multi-walled carbon nanotubes. To measure nephrotoxicity of pristine MWCNTs they used peripheral hemograms, evaluation of the coagulation system, kidney histopathology, and calculating inflammatory cytokines. However, there were no signs of nephrotoxicity, even though the dose of CNTs was higher than in the following studies [94]. The effect of different types of MWCNTs administered intravenously to healthy mice kidneys was also investigated. Results revealed that a higher degree of ammonium modification on the surface of the nanotubes resulted in less accumulation in tissues. In addition, histological analysis 24 h after administration showed no change and no accumulation was observed for all types of MWCNTs tested [95].

## 6. Dermal Toxicity

Skin is the biggest organ in the body, and it can play an important role in drug delivery. As it is a surficial organ, it has a high risk of exposure to carbon nanotubes, since it has the potential to be a major route of exposure during manufacturing, use, or disposal [96]. Carbon nanotubes can enter through the pores, through the lipid bilayers, can adjust the barrier function of the lipids in the membrane, or can be bound to the material of interest and these can then penetrate together [97]. Carbon nanomaterials, when applied to skin, must first pass through the stratum corneum, the outermost layer of the epidermis, which consists of dead cells. Then, they have to penetrate through the lipid pathway that prevents both penetration of environmental substances and losses of body water by surface evaporation [97]. They must be able to penetrate this complex barrier and pass through vital layers of the epidermis and the connection between the epidermis and the skin to access the capillaries in the papillary layer of the dermis to enter the systemic circulation. An illness or condition that causes damage to the stratum corneum may bear them protective functions [98]. The size of the CNTs used is about 100–200 nm in length and 2 nm in diameter; therefore, the CNTs can penetrate through the skin, or they may have some effect on skin penetration of compounds [99]. So far, there have been only a very few publications demonstrating toxic effects of carbon nanotubes on skin (Table 4). Ema et al. studied two different products of pristine MWCNTs and SWCNTs with different physicochemical properties. To test dermal irritation, they used CNT-paste, which was evenly spread on a lint cloth and applied on the skin. Dermal irritation and skin sensitization experiments were made to test toxicity, which showed that one product of MWCNTs was a very weak acute irritant to the skin [96]. Research by Murray et al. on the toxicity of carbon nanotubes has shown that the action of nanotubes when applied to the skin causes oxidative stress in mice, reduction of glutathione concentration, and oxidation of thiol groups [100]. Mice were exposed to SWCNTs at various doses for five consecutive days, and changes were observed. The changes that occurred in rodents resulted in an increase in the number of dermal cells and significant skin thickening [100].

## 7. Cardiovascular Toxicity

Carbon nanotubes can enter the body through the respiratory tract, digestive tract, and by skin contact. Due to their small size and high permeability, CNTs can penetrate biological barriers and enter the blood. They can also enter the body’s circulation directly by injection into a vein or by surgery, thus reaching the heart [101]. Studies reported that ultrafine particles can translocate from the lungs to the systemic circulation by crossing the alveolar–capillary barrier [102]. Salehcheh et al. showed in their study that MWCNTs reduced heart mitochondria viability via inhibition of complex II activity. Carbon nanotubes increased reactive oxygen species generation and lipid peroxidation. This study revealed that oxidative stress is the main mechanism of mitochondrial toxicity in MWCNTs, however, apoptosis and other cytotoxic pathways may also be involved in MWCNT toxicity [103]. Chen et al. showed that MWCNTs can enter the lungs of rats and penetrate the lung blood−gas barrier, which would lead to their bioaccumulation in the liver, kidney, and spleen. Their results also indicated that respiratory exposure of carbon nanotubes influence the cardiovascular system by significantly increasing endothelin-1 (ET-1), angiotensin-converting enzyme (ACE), fibrinogen, and C-reactive protein (CRP) levels. To evaluate cardiotoxicity, they applied electrocardiogram monitoring, histopathological examination, and biochemical analysis [104]. Legramante et al. studied cardiac automatic regulation of pristine single-walled carbon nanotubes after intratracheal instillation. Arterial pressure, baroreflex sequences, and heart rate were analyzed to asses toxicity, which have shown that SWCNTs may affect the autonomic control of cardiac activity [105] (Table 5).

## 8. Neurotoxicity

Due to their nano size, carbon nanotubes can reach many organs and systems, causing toxic effects [107]. Some of them are able to penetrate the blood–brain barrier (BBB) and accumulate in several areas of the central nervous system [108]. The blood–brain barrier is an endothelial barrier lining the brain microvasculature, which provides homeostasis and protection from pathogens, but it can also prevent the penetration of therapeutic drugs into diseased brain tissue [109]. Publications on the neurotoxicity of carbon nanotubes in rodent models are summarized in chronological order in Table 6. In Yang et al., the study of the biodistribution of SWCNTs using intravenous injection revealed that pristine SWNTs were distributed in the brain over the 28 days of the experimental period, meaning nanotubes could overcome the blood–brain barrier to enter into the brain [78]. Cerebrovascular inflammation increases permeability of the BBB [110]. Under inflammatory conditions, tight connections between endothelial cells can become destabilized, causing the blood–brain barrier to become permeable and allowing inflammatory molecules to pass through it, activating neuroglial cells [111]. Functionalized CNTs have been used to deliver drugs, proteins, and nucleic acids into cells, and both in vitro and in vivo studies have demonstrated the neurotoxicity of selected particles that induced inflammation of the nervous system and cognitive impairment [108,112,113]. In in vitro studies, they found that MWCNTs induced DNA damage and overproduction of reactive oxygen species in neuronal cells, leading to a pro-inflammatory response. The inflammation of neurons was confirmed by increased levels of tumor necrosis factor-α (TNFα), interleukin-1β (IL-1β), interleukin-6 (IL-6), and cytokines. For their research, Visalli et al. used differentiated SH-SY5Y cells as an in vitro cell model resembling neuronal cells. Then, to assess neurotoxic and neuroinflammation effects of MWCNTs, they tested cell viability, ROS production, mitochondrial function, as well as DNA damage. [113]. Until now, there have only been a few studies on neurotoxicity of carbon nanotubes in vivo (Table 6). Samiei et al. tested brain toxicity during whole-body exposure after inhalation of pristine multi-walled carbon nanotubes. For this evaluation, they performed mitochondrial evaluation from the hippocampus, frontal cortex, and cerebellum and interpreted parameters of mitochondrial toxicity such as mitochondrial succinate dehydrogenase activity, generation of reactive oxygen species (ROS), mitochondrial membrane potential collapse, mitochondrial swelling, cytochrome c release, ATP level, mitochondrial reduced glutathione, and lipid peroxidation [114]. Carbon nanotubes were shown to cause toxic effects, which could be a reason of apoptosis signaling, and as a consequence could be a major pathology of neurodegenerative diseases [114]. In studies presented by Aragon et al., pristine multi-walled carbon nanotubes were administrated through oropharyngeal aspiration, which led to a BBB-depended neuroinflammatory response. Neurotoxicity was stated by increased inflammatory marker transcription, which indicated blood–brain barrier disruption and neuroinflammation [111].

## 9. Prospects on Standardized In Vivo Testing

One of the biggest problems for investigating toxicological outcomes is growing numbers of carbon nanotubes, such as SWCNTs, MWCNTS, and DWCNTs, with a variety of properties or functionalization. A wide range of carbon nanotubes makes it very challenging for toxicological testing of a specific material. In order to be able to compare the results of toxicity tests, the same test conditions should be applied.

Nowadays, there are current guidelines for safety assessments of nanoparticles, which give some recommendation for preparation and toxicity testing of nanomaterials. Some of the documents are the *Guidance Manual for the Testing of Manufactured Nanomaterials* [115], The Organisation for Economic Co-operation and Development (OECD) *Guidance on Sample Preparation and Dosimetry for the Safety Testing of Manufactured Nanomaterials* [116], or, more specific, the *Ecotoxicology and Environmental Fate of Manufactured Nanomaterials: Test Guidelines* [117]. OECD ensures development and updates of test guidelines so they can give the most important information for what procedures should be used for the majority of upright toxicological evaluation. Testing guidelines focus on physical-chemical properties, environmental fate, inhalation toxicity, genotoxicity, and toxicokinetics.

OCED recommends some physical-chemical data, which are relevant for identifying or characterizing carbon nanotubes, such as degree of purities, basic morphology, description of surface chemistry, catalytic activity, porosity, and many more. Different research of carbon nanotube toxicity uses individual methods of preparation of dispersion, which can lead to distinction in CNT toxicity. For that reason, there is a need to standardize dispersion methods during the preparation process. Conditions such as pH, addition of compounds, or using procedures such as ultrasonification may improve dispersion of suspension [118]. There are few nanomaterial dispersion protocols that identify parameters that should be taken into consideration, such as carbon nanotube properties, stock concentration, volume of dispersion medium, stabilizing agents, or dispersion procedure [119].

An important topic when discussing nanoparticles is data. For the majority of research, all the information and knowledge are used during the time of the research. The EU NanoSafety Cluster states the importance of data logging to obtain exploitable data [120]. One storage is ISA-TAB-Nano, which allows us to represent and share information about nanomaterials, small molecules, and biological specimens along with their assay characterization data, one of which is about mammalian toxicology in vivo [121].

## 10. Conclusions

Carbon nanotubes, due to their unique properties, may serve as a very attractive material used in medicine, and can be used as drug carriers, contrast agents, or biological platforms. They can also be easily provided with strong photothermal properties and allowed to avoid the metallic core. Toxicity of CNTs results from interactions with biological systems and induction of toxic reactions. Moreover, their harmfulness depends on both the physicochemical properties, applied concentration, as well as the routes of exposure. Studies showed that the main mechanisms responsible for harmful effects are oxidative stress, disruption of cellular compartments, and inflammation. Even though carbon nanotubes seem to exhibit hazardous properties, it does not necessarily mean they will not be used in nanomedicine. With the use of selective targeting, pristine carbon nanotubes could reach damaged tissues. What is also important is that functionalization of pristine CNTs can reduce or even eliminate their toxicity. In this short review, the latest information regarding toxicity osf pristine CNTs under in vivo conditions focusing on rats and mice were compiled and described. Evaluation of toxicity of carbon nanotubes differs in all research, so the comparison of obtained results between studies is difficult. Diversity concerns not only animal models, but also administration manner, and concentration and properties of CNTs. After analyzing data of this review, we can notice that some changes must be done in the future of toxicological assessment of this nanomaterial. Firstly, carbon nanotubes in the tests should have a proper physicochemical characterization, even if the manufacturer provides such information. Carbon nanotubes vary by company as well as by time of manufacturing, making direct comparisons between studies difficult. Establishing criteria for CNT preparation and dispersion also plays a key role in determining data comparability. Exposure should also be carefully monitored, as particles tend to aggregate and proper dispersion in solution may be difficult or even impossible. Careful assessment of all aspects should be considered, including dosing and post-exposure time. Another important view is the number of experimental groups and animals per group. CNT toxicity studies vary with the number of animals, and there must be an adequate, well-defined quantity to reliably interpret the data. Due to significant changes between carbon nanotubes, it is difficult to understand exactly which aspects of carbon nanotubes, e.g., surface area, mass concentrations, lengths, or a combination of these characteristics, affect their toxicity. So far, there have only been a few publications on long-term toxicity, since most of them focus on acute effects. For the display of some harmful effects, there is a need for a certain amount of time to pass since exposure for symptoms to develop. This should be taken into consideration, since short- and long-term safety concerns make it difficult to go on with clinical use. Since the majority of introduced studies focus on different toxic aspects and it is hard to compare them, we are convinced there is a need for strict guidelines. Using existing recommendations and the libraries for nanoparticle safety, we are able to receive more data when synthetizing new CNTs. Carbon nanotubes have an immense potential for biomedical applications, such as in the fabrication of sensors, drug targeting, cancer treatment or antimicrobial activity, so if the clinical applications are considered, safety of CNTs must be shown in reasoning and fulfilling experiments.

## Figures and Tables

**Table 1:** Pulmotoxicity of pristine carbon nanotubes using rodents as animal models.

References	Materials	Materials Properties	Animals	Routes of Administration	Doses/Concentrations	Exposure Times	Toxicity Effects
Hojo et al., 2022 [32]	MWCNTs	Length 5.11 µm, width 84.7 nm	Fisher 344 rats	Intratracheal instillation	0, 0.125, and 0.5 mg/kg	Every 4 weeks for over 2 years	Dose- and time-dependent inflammatory, fibrotic, and hyperplastic lesions. In the high-dose group, significantly increased incidences of lung carcinomas, lung adenomas, and pleural mesotheliomas.
Fujita et al., 2022 [57]	MWCNTs	Diameter 9.5 nm, length 1.5 μm,	Crl:CD rats	Intratracheal instillation	0.25, 0.5 and 1.0 mg/kg	1, 3, 7, 30, 90, and 180 days	BALF cells, total protein, lactate dehydrogenase, and pro-inflammatory cytokines showed that MWCNTs induced pneumonia.
Numano et al., 2021 [69]	MWCNTs	Diameter 75 nm, length 9 μm	Fisher 344/DuCrlCrlj rats	Intratracheal instillation	0.2, 0.4 and 0.8 mg/kg	6 weeks	Increased lung weight, inflammation in the lungs, fibrosis of the visceral and parietal pleura.
Seidel et al., 2021 [63]	MWCNTs	Diameter 67 and 12 nm, length 4 and 0.4 μm, purity > 95%	Sprague Dawley rats	Nose-only inhalation	0.5 and 1.5 mg/m3	4 weeks	Induction of pneumonia, dose-dependent increase in the number of genes and proteins with differential expression.
Kim et al., 2020 [74]	MWCNTs	Diameter 5–10 nm, length > 1 µm	Sprague Dawley rats	Nose-only inhalation	0, 0.257, 1.439 and 4.253 mg/m^3^	1, 7, and 28 days	Increase in lung inflammation parameters in BALF.
Lim et al., 2020 [61]	MWCNTs	Diameter 56.0–61.0 nm, length 4.08–4.88µm	B6C3F1 mice	Pharyngeal aspiration	40 µg/mouse	1, 7, and 28 days	Induction of high levels of leukotriene B4 and prostaglandin E2, acute inflammation in the lungs at low doses.
El-Gazzar et al., 2019 [67]	DWCNTs MWCNTs	Diameter 1–3 nm Diameter 55 nm, length 6.5 μm	Fisher 344/DuCrlCrlj rats	Intratracheal intrapulmonary spraying (TIPS)	0.25 and 0.5 mg/rat	1 and 6 weeks	DWCNTs have a lower degree of pulmonary toxicity and imperceptible pleural toxicity than MWCNTs.
National Toxicology Program, 2019 [75]	MWCNTs	Diameter 10–20 nm, length 10–30 µm	Sprague Dawley rats, B6C3F1/N mice	Whole-body inhalation	0, 0.1, 0.3, 1, 3, and 10 mg/m^3^	14, 42, or 126 days	There was a brown discoloration of the lungs, enlarged and congested bronchial and mediastinal lymph nodes. Increased incidence of chronic inflammation in the lungs.

**Table 2:** Hepatotoxicity of pristine carbon nanotubes using rodents as animal models.

References	Materials	Materials Properties	Animals	Routes of Administration	Doses/ Concentrations	Exposure Times	Toxicity Effects
Kim et al., 2015 [74]	MWCNTs	Diameter 12.5 ± 2.5 nm, purity > 95%	C57BL/6J mice	Intratracheal instillation	0.1 mg/mouse	1 year	Mice developed nonalcoholic steatohepatitis-like phenotype, characterized by inflammation, hepatic steatosis, and fibrosis.
Lin et al., 2013 [83]	SWCNTs	Diameter 0.8–1.2 nm, length of several microns	Wistar rats	Intratracheal instillation	7.5, 15, and 22.5 mg/kg	15 days	Rise in extract concentrations of choline and phosphocholine, together with decreased lipids and lipoproteins, which indicated a disruption of membrane fluidity caused by lipid peroxidation.
Folkman et al., 2009 [68]	SWCNTs	Diameter 0.9–1.7 nm, lengths < 1 µm	Fisher 344 rats	Intragastric administration	0.064 and 0.64 mg/kg	Rats were sacrificed at 9 weeks of age	SWCNT increased the levels of 8-oxodG in liver, which is likely to be caused by genotoxicity.
Yang et al., 2008 [87]	SWCNTs	Diameter 10–30 nm, length 2–3 μm, purity > 95%	CD-ICR mice	Tail-vein injections	40, 200 μg/mouse and 1.0 mg/mouse	3 months	Low hepatotoxicity compared to functionalized SWCNTs.
Kasai et al., 2016 [70]	MWCNTs	Diameter 95.5–109.6 nm, length 5.8–5.9 μm.	Fisher 344 rats	Whole-body inhalation	0, 0.02, 0.2, and 2 mg/m^3^	6 h/day, 5 days/week for 104 weeks	Lung carcinomas were significantly increased in animals exposed to MWCNTs, however, there was no development of pleural mesothelioma.
Folkman et al., 2009 [68]	SWCNTs	Diameter 0.9–1.7 nm, length < 1 µm	Fisher 344 rats	Intragastric administration	0.064 and 0.64 mg/kg	Rats were sacrificed at 9 weeks of age	Increased levels of 8-oxodG in the liver, which are likely to be caused by genotoxicity.
Ryman-Rasmussen et al., 2009 [65]	MWCNTs	Diameter 10–50 nm, length 0.5–50 μm	C57BL6 mice	Nose-only inhalation	1 and 30 mg/m^3^	1 day, 2 weeks, 6 weeks, or 14 weeks	Increase in subpleural fibrosis.
Mitchell et al., 2007 [66]	MWCNTs	Diameter 10–20 nm, length 5–15 μm, purity > 95%	C57BL/6 mice	Whole-body inhalation	0.3, 1 and 5 mg/m^3^	7 and 14 days	Inflammation and histopathological changes in the lungs were not observed.
Shvedova et al., 2005 [62]	SWCNTs	Diameter 1– 4 nm, surface area 1040 m^2^/g	C57BL/6 mice	Pharyngeal aspiration	0, 10.0, 20.0 and 40.0 μg/mouse	1, 3, 7, 28, and 60 days	There was acute inflammation and, over time, pulmonary fibrosis.
Lam et al., 2004 [59]	CNTs	-	B6C3F mice	Intratracheal instillation	0.1 and 0.5 mg/mouse	7 and 90 days	Mortality occurred in mice that received 0.5 mg of CNTs.
Warheit et al., 2003 [60]	SWCNTs	Diameter 1.4 nm, length > 1 μm	Crl:CD(SD)IGS BR rats	Intratracheal instillation	1 and 5 mg/kg	24 h, 1 week, 1 month, and 3 months	Initial mortality in 15% of rats, there were transient inflammation and multifocal granulomas, irrespective of the dose.

**Table 3:** Nephrotoxicity of pristine carbon nanotubes using rodents as animal models.

References	Materials	Materials Properties	Animals	Routes of Administration	Doses/ Concentrations	Exposure Times	Toxicity Effects
Zamani et al., 2021 [91]	MWCNTs	Diameter 10 and 100 nm, length 140–180 μm	Wistar rats	Inhalation	5 mg/m^3^	2 weeks	Mitochondrial damage in kidney cells, including renal tubular cells.
Awogbindin et al., 2021 [92]	MWCNTs	Diameter 15–30 nm, length 15–20 μm, purity > 95%	Wistar rats	Intravenous administration	1 mg/kg	15 days	Severe disseminated congestion and infiltration of inflammatory cells in the kidneys.
Guzmán-Mendoza et al., 2020 [93]	CNTs	Diameter 20–40 nm, length 30 μm	BALB/c mice	Tail-vein injections	2 mg/kg	1, 14, 29, and 60 days	Pristine CNTs have the highest toxicity due to accumulation in the kidneys and the lungs even at 60 days; moreover, they produced lung damage, tumor growth, hepatotoxicity, renal failure, and could possibly induce heart failure.
Tang et al., 2012 [94]	MWCNTs	Diameter 10–20 nm, length 5–50 μm, purity > 95%	Kunming mice	Tail-vein injections	100 mg/mouse	1 day, 3 days, and 1 year	There were no differences in vivo in inflammatory responses, the coagulation system, hemograms, or vital kidney functions.
Jain et al., 2011 [89]	MWCNTs	Diameter 100–500 nm, length < 500 nm	Swiss albino mice	Tail-vein injections	10 mg/kg	7 and 28 days	No apparent nephrotoxicity.

**Table 4:** Dermal toxicity of pristine carbon nanotubes using rodents as animal models.

References	Materials	Materials Properties	Animals	Routes of Administration	Doses/Concentrations	Exposure Times	Toxicity Effects
Ema et al., 2011 [96]	MWCNTs and SWCNTs	Diameter 1.8, 3, 44, and 60 nm	Kbl:NZW rabbits, Slc:Hartley guinea pigs	Topical administration	5 mg/mouse, 10 mg/mouse	1, 24, 48 and 72 h	One MWCNT caused very slight erythema at 24 h, but not at 72 h, after patch removal in the dermal irritation experiments, very weak acute irritant to the skin and eyes.
Murray et al., 2009 [100]	SWCNTs	-	SKH-1 Hairless mice	Topical administration	40 g/mouse, 80 g/mouse, or 160 g/mouse	5 days	SWCNTs induced free radical generation, oxidative stress, and inflammation, thus causing dermal toxicity.

**Table 5:** Cardiac toxicity of pristine carbon nanotubes using rodents as animal models.

References	Materials	Materials Properties	Animals	Routes of Administration	Doses/Concentrations	Exposure Times	Toxicity Effects
Chen et al., 2015 [104]	MWCNTs	Diameter < 8 nm, length 0.5–2 μm	Wistar-Kyoto rats	Intratracheal instillation	600 μg/kg	7 and 30 days	MCWNTs caused subchronic toxicity, especially the sustained inflammation of the pulmonary and cardiovascular system.
Legramante et al., 2009 [105]	SWCNTs	Diameter 1.2–1.6 nm, length 2–5 nm, and surface area 300 m^2^/g	Wystar-Kyoto rats	Intratracheal instillation	1 μg/g	24 h, 2 weeks, 24 weeks.	SWCNTs may alter the TFIIB-related factor, thus affecting the autonomic cardiovascular control regulation.
Li et al., 2007 [106]	SWNCTs	Diameter 1–4 nm, surface area 1040 m^2^/g	C57BL/6 mice	Intrapharyngeal instillation	10 and 40 µg/mouse	7, 28, and 60 days	SWCNTs induced activation of heme oxygenase-1, damage to mtDNA, and acceleration of the formation of atherosclerotic plaques.

**Table 6:** Neurotoxicity of pristine carbon nanotubes using rodents as animal models.

References	Materials	Materials Properties	Animals	Routes of Administration	Doses/Concentrations	Exposure Times	Toxicity Effects
Samiei et al., 2020 [114]	MWCNTs	Diameter 10 and 100 nm, length 0.14–1.7 and 0.16–1.8 μm	Wistar rats	Inhalation	5 mg/m^3^	2 weeks	MWCNTs induce damage in varying degrees on the mitochondrial respiratory chain and increase mitochondrial ROS formation in different parts of rat brains.
Aragon et al., 2017 [111]	MWCNTs	-	C57BL6 mice	Oropharyngeal aspiration	10 and 40 μg/mouse	4 h	Acute pulmonary exposure to MWCNTs causes neuroinflammatory responses that are dependent on the disruption of BBB integrity.

## Data Availability

Not applicable.
